# Stage-Related Defense Response Induction in Tomato Plants by *Nesidiocoris tenuis*

**DOI:** 10.3390/ijms17081210

**Published:** 2016-07-27

**Authors:** Mario Naselli, Alberto Urbaneja, Gaetano Siscaro, Josep A. Jaques, Lucia Zappalà, Víctor Flors, Meritxell Pérez-Hedo

**Affiliations:** 1 Dipartimento di Agricoltura, Alimentazione e Ambiente (Di3A), University of Catania, Via Santa Sofia 100, 95123 Catania, Italy; marionaselli13@gmail.com (M.N.); gsiscaro@unict.it (G.S.); lzappala@unict.it (L.Z.); 2 Unidad Asociada de Entomología UJI-IVIA, Centro de Protección Vegetal y Biotecnología, Instituto Valenciano de Investigaciones Agrarias (IVIA), Carretera de Moncada-Náquera Km. 4.5, Moncada, 46113 Valencia, Spain; aurbaneja@ivia.es (A.U.); mperezh@ivia.es (M.P.-H.); 3 Unitat Associada d’Entomologia UJI-IVIA, Departament de Ciències Agràries i del Medi Natural, Universitat Jaume I, UJI, Campus del Riu Sec, 12071 Castelló de la Plana, Spain; josep.jaques@uji.es (J.A.J.); flors@uji.es (V.F.)

**Keywords:** *Bemisia tabaci*, *Encarsia formosa*, tomato, induced plant response, indirect defense, phytohormones

## Abstract

The beneficial effects of direct predation by zoophytophagous biological control agents (BCAs), such as the mirid bug *Nesidiocoris tenuis*, are well-known. However, the benefits of zoophytophagous BCAs’ relation with host plants, via induction of plant defensive responses, have not been investigated until recently. To date, only the females of certain zoophytophagous BCAs have been demonstrated to induce defensive plant responses in tomato plants. The aim of this work was to determine whether nymphs, adult females, and adult males of *N. tenuis* are able to induce defense responses in tomato plants. Compared to undamaged tomato plants (i.e., not exposed to the mirid), plants on which young or mature nymphs, or adult males or females of *N. tenuis* fed and developed were less attractive to the whitefly *Bemisia tabaci*, but were more attractive to the parasitoid *Encarsia formosa*. Female-exposed plants were more repellent to *B. tabaci* and more attractive to *E. formosa* than were male-exposed plants. When comparing young- and mature-nymph-exposed plants, the same level of repellence was obtained for *B. tabaci*, but mature-nymph-exposed plants were more attractive to *E. formosa*. The repellent effect is attributed to the signaling pathway of abscisic acid, which is upregulated in *N. tenuis*-exposed plants, whereas the parasitoid attraction was attributed to the activation of the jasmonic acid signaling pathway. Our results demonstrate that all motile stages of *N. tenuis* can trigger defensive responses in tomato plants, although these responses may be slightly different depending on the stage considered.

## 1. Introduction

Plants are able to defend themselves from arthropods, pathogens and, in general, from biotic and abiotic stress conditions [1,2,3]. To this end, plants activate a cascade of events that include transcriptome changes of some of the genes involved in the biosynthesis of phytohormones that lead—directly and indirectly—to defensive responses [4,5]. The main phytohormones responsible for these responses are jasmonic, salicylic, abscisic acids, and ethylene (JA, SA, ABA, and ET respectively) [1,2,3,6,7,8]. Depending on the herbivore’s feeding habits (chewing, phloem, or cell content feeders), different hormone-related signaling pathways are triggered [9,10,11,12,13]. For instance, it is known that insects with piercing-sucking mouthparts (especially phloem feeders like most of the Hemiptera) mostly induce the SA-mediated resistance pathway, whereas insects with chewing mouthparts predominantly trigger the JA pathway [14,15,16]. However, JA may also be induced by cell content feeders such as thrips (Thysanoptera: Thripidae) and spider mites (Acari: Tetranychidae) [8]. 

Zoophytophagous arthropods—which feed both on other arthropod as prey and on plants during the same developmental stages—can also activate the same defense mechanisms as strict herbivores [17,18,19,20,21]. It is well-known that zoophytophagy provides adaptive advantages, such as the ecological flexibility to consume both prey and plants, thereby allowing the survival of these predators on plants when prey is scarce [22,23,24]. This is the case for *Nesidiocoris tenuis* Reuter (Hemiptera: Miridae), a widely used biological control agent (BCA) which has been extremely effective in controlling some key tomato pests, including the tobacco whitefly *Bemisia tabaci* (Gennadius) (Hemiptera: Aleyrodidae) and the invasive South American pinworm *Tuta absoluta* (Meyrick) (Lepidoptera: Gelechiidae) [25,26,27]. Apart from this predation-dependent beneficial effect, the activity of *N. tenuis* females (feeding and/or oviposition) on tomato plants activates the ABA and JA pathways, which make tomato plants less attractive to phytophagous *B. tabaci* and more attractive to the whitefly parasitoid *Encarsia formosa* (Gahan) (Hymenoptera: Aphelinidae), respectively [21]. In addition, herbivore-induced plant volatiles (HIPVs) from *N. tenuis*-exposed plants can induce plant defenses in neighboring, undamaged (not exposed to the mirids) plants via JA, which result in the attraction of parasitoids [21]. These effects on plant defensive responses might be a reasonable explanation of the achievement reached by *N. tenuis* in integrated pest management programs in tomatoes. 

Pérez-Hedo, et al. [20] showed that the females of three different zoophytophagous BCAs (*N. tenuis*, *Macrolophus pygmaeus* Rambur, and *Dicyphus maroccanus* Wagner) differ in their ability to induce defensive responses in tomato plants, resulting in varying degrees of attractiveness of the plants to pests and natural enemies. In the case of tomato plants exposed to and therefore presumably punctured by *N. tenuis*, these plants were less attractive to the whitefly *B. tabaci* and to the lepidopteran *T. absoluta*. In contrast, tomato plants exposed to *M. pygmaeus* and *D. maroccanus* were not able to repel *B. tabaci* and, more interestingly, became more attractive to *T. absoluta*. Pappas, et al. [28] showed that tomato plants exposed to adult females, fifth instar nymphs, and young virgin females of *M. pygmaeus* were able to induce plant resistance against the two-spotted spider mite *Tetranychus urticae* Koch (Acari: Tetranychidae). In tomato plants infested by *T. urticae*, the number of *T. urticae* eggs laid was lower when these tomato plants had been exposed previously to *M. pygmaeus* [28]. 

To date, it has been shown only that the feeding and oviposition activities of *N. tenuis* adult females induce defensive plant responses in tomato plants [20,21]. Nevertheless, under field conditions, it is usually common to find a mix of instars and/or stages of this and other mirids [29]. To know whether nymphal instars and males are also able to induce defensive plant responses, in this work we evaluated the response induced by the feeding (plus oviposition in the case of adult females) activity of different instars/stages of *N. tenuis* compared to undamaged plants. This response was assessed by means of behavioral bioassays in a Y-tube olfactometer using adults of the herbivore *B. tabaci* and of the entomophagous parasitoid *E. formosa*. In addition, ultra-performance liquid chromatography coupled to mass spectrometry (UPLC-MS) and quantitative gene expression of selected phytohormones and genes, were analyzed.

## 2. Results

### 2.1. Olfactory Responses Induced by N. tenuis-Punctured Plant

**Figure 1:**
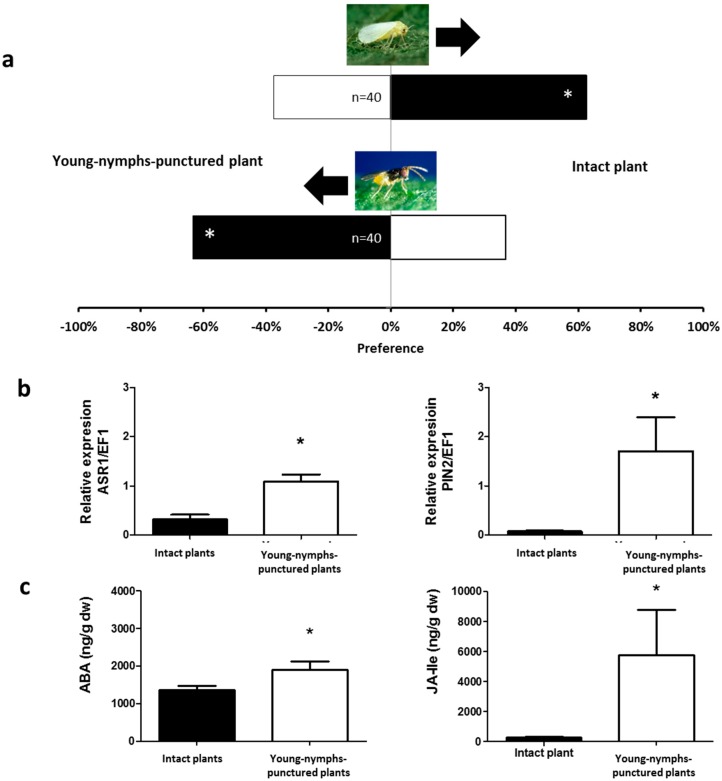
Plant responses induced by young *N. tenuis* nymphs. (**a**) Response of *B. tabaci* and *E. formosa* females when they were given the choice between intact tomato plants and punctured tomato plants in a Y-tube olfactometer. Significant differences using χ^2^ test, *p* < 0.05 are marked with (*); (**b**) Expression of the defensive genes *ASR1* and *PIN2* (target genes induced by the phytohormones abscisic acid (ABA) and jasmonate-isoleucine (JA-Ile), respectively). Data are presented as the mean of the ratio between the concentration of the gene transcripts and that of the constitutive elongation factor 1 (*EF1*) gene. Significant differences were obtained by comparing punctured plants to intact plants. Results from a one-tailed *t*-test are marked with (*) (*p* < 0.05); (**c**) ABA and JA-Ile levels in the apical part of tomato plants. Each of the presented results is the mean of the hormone concentration (ng/g) of five independent analyses ± SE (*n* = 5). Significant differences were obtained by comparing punctured plants to intact plants. Results from a one-tailed *t*-test are marked with (*) (*p* < 0.05).

**Figure 2:**
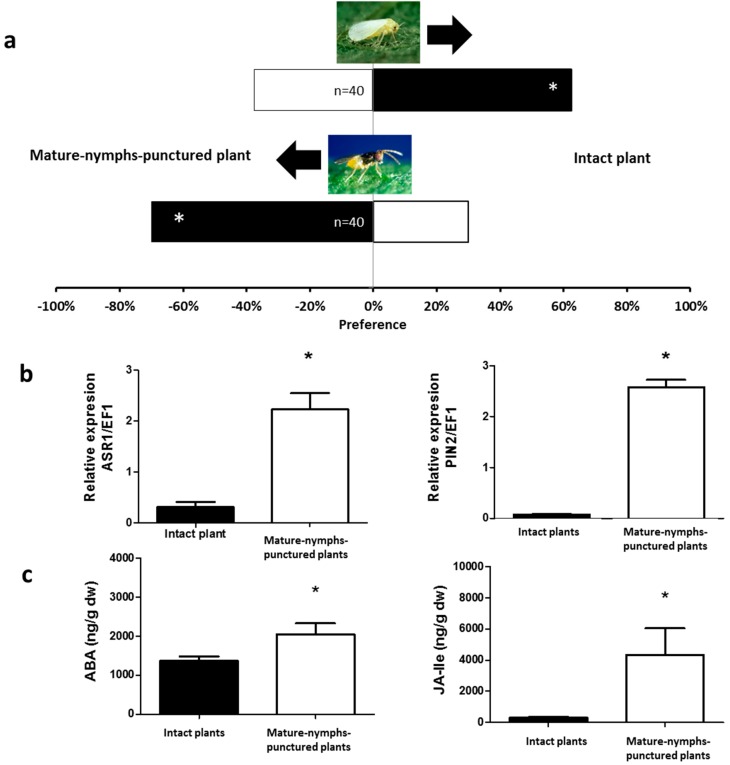
Plant responses induced by mature *N. tenuis* nymphs. (**a**) Response of *B. tabaci* and *E. formosa* females when were given the choice between intact tomato plants and punctured tomato plants in a Y-tube olfactometer. Significant differences using χ^2^ test, *p* < 0.05 are marked with (*); (**b**) Expression of the defensive genes *ASR1* and *PIN2* (target genes induced by the phytohormones ABA and JA-Ile, respectively). Data are presented as the mean of the ratio between the concentration of the gene transcripts and that of the constitutive *EF1* gene. Significant differences were obtained by comparing punctured plants to intact plants. Results from one tailed *t*-test are marked with (*) (*p* < 0.05); (**c**) ABA and JA-Ile levels in the apical part of tomato plants. Each of the presented results is the mean of the hormone concentration (ng/g) of five independent analyses ± SE (*n* = 5). Significant differences were obtained by comparing punctured plants to intact plants. Results from a one tailed *t*-test are marked with (*) (*p* < 0.05).

When plants exposed to young (NI and NII) or mature nymphs (NIV and NV) of *N. tenuis* were compared to undamaged plants (i.e., not exposed to the mirid), a clear preference of *B. tabaci* for undamaged plants was observed (*χ*^2^ = 5.000, *p* = 0.0253 and *χ*^2^ = 7.200, *p* = 0.0073, respectively, Figure 1a and Figure 2a).

**Figure 3:**
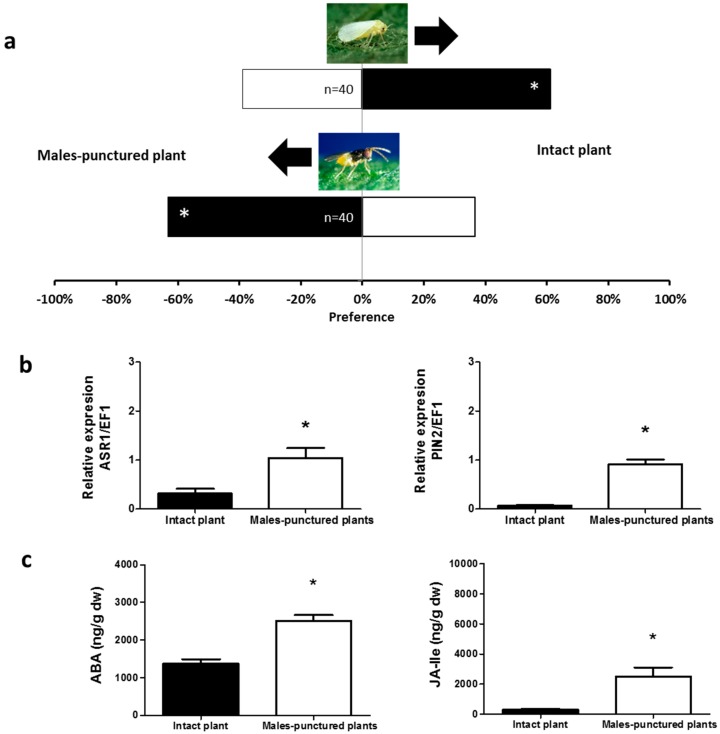
Plant responses induced by *N. tenuis* males. (**a**) Response of *B. tabaci* and *E. formosa* females when were given the choice between intact tomato plants and punctured tomato plants in a Y-tube olfactometer. Significant differences using χ^2^ test, *p* < 0.05 are marked with (*); (**b**) Expression of the defensive genes *ASR1* and *PIN2* (target genes induced by the phytohormones ABA and JA-Ile, respectively). Data are presented as the mean of the ratio between the concentration of the gene transcripts and that of the constitutive *EF1* gene. Significant differences were obtained by comparing punctured plants to intact plants. Results from a one-tailed *t*-test are marked with (*) (*p* < 0.05); (**c**) ABA and JA-Ile levels in the apical part of tomato plants. Each of the presented results is the mean of the hormone concentration (ng/g) of five independent analyses ± SE (*n* = 5). Significant differences were obtained by comparing punctured plants to intact plants. Results from a one-tailed *t*-test are marked with (*) (*p* < 0.05).

**Figure 4:**
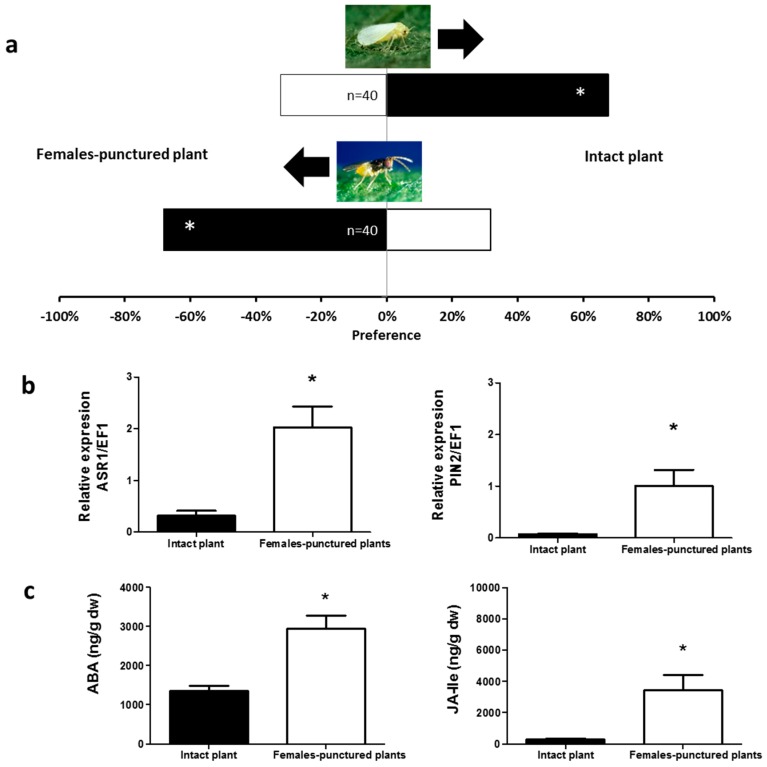
Plant responses induced by *N. tenuis* females. (**a**) Response of *B. tabaci* and *E. formosa* females when were given the choice between intact tomato plants and punctured tomato plants in a Y-tube olfactometer. Significant differences using χ^2^ test, *p* < 0.05 are marked with (*); (**b**) Expression of the defensive genes *ASR1* and *PIN2* (target genes induced by the phytohormones ABA and JA-Ile, respectively). Data are presented as the mean of the ratio between the concentration of the gene transcripts and that of the constitutive *EF1* gene. Significant differences were obtained by comparing punctured plants to intact plants. Results from a one-tailed *t*-test are marked with (*) (*p* < 0.05); (**c**) ABA and JA-Ile levels in the apical part of tomato plants. Each of the presented results is the mean of the hormone concentration (ng/g) of five independent analyses ± SE (*n* = 5). Significant differences were obtained by comparing punctured plants to intact plants. Results from a one-tailed *t*-test are marked with (*) (*p* < 0.05).

Similarly, undamaged tomato plants were more attractive for *B. tabaci* than tomato plants exposed to either adult females or males of *N. tenuis* (*χ*^2^ = 9.800, *p* = 0.0017 and *χ*^2^ = 3.951, *p* = 0.0468, respectively, Figure 3a and Figure 4a). Unlike *B. tabaci*, all plants exposed to the mirids were found to be more attractive to the parasitoid *E. formosa* than to undamaged plants (*χ*^2^ = 4.267, *p* = 0.0389 for young nymphs—Figure 1a; *χ*^2^ = 9.600, *p* = 0.0019 for mature nymphs—Figure 2a; *χ*^2^ = 6.898, *p* = 0.0086 for adult males—Figure 3a; and *χ*^2^ = 11.640, *p* = 0.0006 for adult females—Figure 4a).

**Figure 5:**
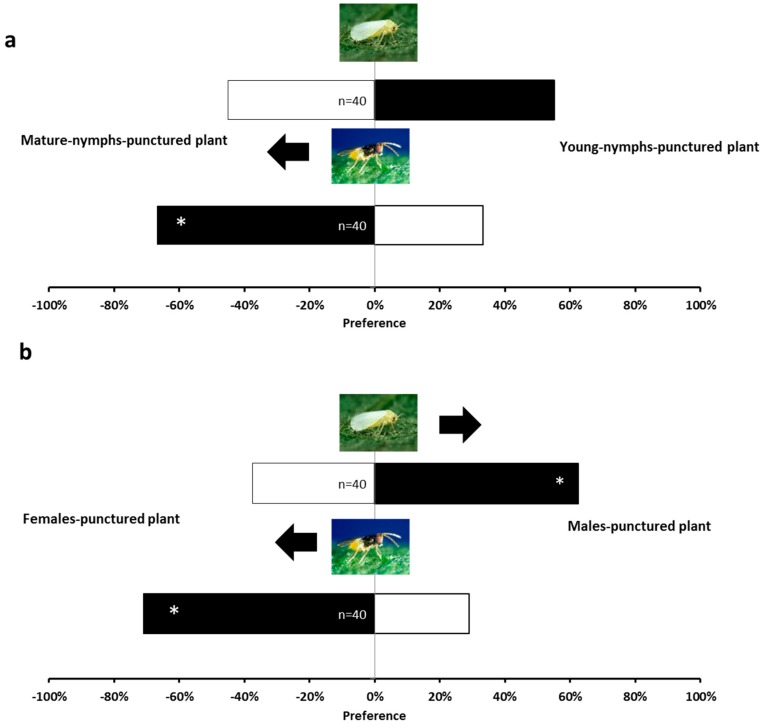
Response of *B. tabaci* and *E. formosa* females in a Y-tube olfactometer. (**a**) Comparison between mature-nymph-punctured tomato plants with young-nymph-punctured tomato plants; (**b**) Comparison between female-punctured tomato plants with male-punctured tomato plants. Significant differences using χ^2^ test, *p* < 0.05 are marked with (*).

When adult-female-exposed plants were compared with plants exposed to adult males, the former were more repellent to *B. tabaci* (*χ*^2^ = 5.000, *p* = 0.0253) and more attractive to *E. formosa* (*χ*^2^ = 10.90, *p* = 0.0010, Figure 5b) than those exposed to adult males. However, when plants exposed to either young or mature nymphs were exposed to *B. tabaci*, no significant differences were detected (*χ*^2^ = 1.852, *p* = 0.1736). However, plants exposed to mature nymphs were more attractive to the parasitoid *E. formosa* than those exposed to young nymphs (*χ*^2^ = 6.667; *p* = 0.0098, Figure 5a).

### 2.2. Phytohormones Analysis and Plant Gene Expression

In the apical part of all plants exposed to *N. tenuis*, the endogenous levels of ABA and of the bioactive phytohormone JA-Isoleucine (JA–ILE) were higher than in the apical part of undamaged plants (ABA: *t* = 1.992, *p* = 0.0359 for young nymphs—Figure 1c; *t* = 2.206, *p* = 0.0260 for mature nymphs—Figure 2c; *t* = 6.065, *p* < 0.0001 for males—Figure 3c; *t* = 4.298, *p* = 0.0008 for females—Figure 4c; and jasmonate-isoleucine (JA-Ile): *t* = 1.815, *p* = 0.0498 for young nymphs—Figure 1c; *t* = 2.153, *p* = 0.0272 for mature nymphs—Figure 2c; *t* = 3.711, *p* = 0.0020 for males—Figure 3c; *t* = 3.344, *p* = 0.0037 for females—Figure 4c). Consistent with these observations, the analysis of the relative expression of genes involved in indirect defense showed transcriptional differences between *N. tenuis*-exposed plants and undamaged plants. The *ASR1* gene (a marker for ABA) and the *PIN2* gene (a marker for JA) were upregulated in all *N. tenuis*-exposed plants compared to undamaged plants (*ASR1*: *t* = 4.276, *p* = 0.0010 for young nymphs—Figure 1b; *t* = 5.227, *p* = 0.0003 for mature nymphs—Figure 2b; *t* = 3.239, *p* = 0.0059 for males—Figure 3b; *t* = 3.730, *p* = 0.0023 for females—Figure 4b; and *PIN2*: *t* = 2.374, *p* = 0.0225 for young nymphs—Figure 1b; *t* = 15.65, *p* < 0.0001 for mature nymphs—Figure 2b; *t* = 8.185, *p* < 0.0001 for males—Figure 3b; *t* = 3.033, *p* = 0.0081 for females—Figure 4b).

## 3. Discussion

Our results are the first evidence that all motile stages of *N. tenuis* can induce defensive responses in tomato plants. All motile stages can damage plants by feeding on them, whereas adult *N. tenuis* females may also cause damage through oviposition. Our results also demonstrate the direct relationship between *N. tenuis’* plant feeding and defense induction in tomato. Specifically, the feeding on tomato plants by *N. tenuis* NI, NII, NIV, NV, adult males, and adult females all resulted in reduced attractiveness for *B. tabaci* and enhanced attractiveness for *E. formosa* relative to undamaged plants.

Olfactometer results confirm the positive correlation between ABA concentrations and induced repellence to whiteflies as well as between JA and induced attraction of *E. formosa* [20,21]. Interestingly, the stronger behavioral response observed in plants exposed to *N. tenuis* adult females in the olfactometer could be attributed to chemicals either emitted or triggered by *N. tenuis* eggs inserted into the plant tissues, as occurred in other phytophagous species [19,30]. Therefore, the presence of *N. tenuis* eggs on a plant could cause a synergistic effect with feeding, resulting in enhanced repellence for *B. tabaci* and enhanced attraction of *E. formosa* in olfactory bioassays. In sum, the dual activity of females (i.e., feeding and oviposition) could explain these results. However, further research is needed. Indeed, volatile organic compounds (VOCs) emitted by plants in response to herbivore attack—either by feeding and/or by endo/esophytic oviposition—are known to repel further herbivore attacks [30,31]. Thus, the higher intensity of the effect produced by *N. tenuis* adult females on exposed plants might explain the stronger attraction of *E. formosa*, as previously demonstrated in different biological models [32]. Therefore, the potential presence of egg elicitors has to be better investigated. Another issue is the absence of specific defense responses by the plant to the mirid eggs, but this could be explained by the high degree of adaptation of these mirids to plants, confirming their mutualistic relations and suggesting a co-evolutionary approach to understanding these interactions.

Furthermore, the results suggest that the joint use of different instars of *N. tenuis* under greenhouse conditions is a better implementation strategy with this mirid, because the simultaneous presence of different cohorts avoids strong hormonal fluctuations in tomato plants, thereby reducing the negative impact on harvest.

Abiotic stresses, such as water stress or desiccation, induce the activation of the ABA pathway [33,34,35]. Nevertheless, ABA has been reported both as an inducer of plant defense response to necrotrophic pathogens and as an inhibitor of biotrophic pathogens [8]. Apart from the work of Pérez-Hedo, et al. [21], where high levels of ABA in tomato plants were shown to repel the whitefly *B. tabaci*, little information is available in relation to the effects of ABA on arthropods. JA is known for inducing direct and indirect plant defense responses against arthropods, this phytohormone works together with ET in orchestrating these responses [1,8]. The direct defense consists of the production of secondary metabolites, such as proteinase inhibitors, that inhibit the development of insects on activated plants [36,37], whereas indirect defense has been recently observed in trophic interaction studies. Particularly, this latter phenomenon is due to the production and release of VOCs. The synthesis and emission of VOCs is triggered by JA synthesis and mediates the attraction or rejection of beneficial and phytophagous species, respectively [10,36,38,39]. All the above-mentioned phytohormonal activity for indirect plant defense—which is induced by herbivores—culminates in the production and release of HIPVs [40]. These HIPVs are the signals for plant–plant and plant–arthropod communication, and result in the attraction of natural enemies and the repellence of herbivores, demonstrating the key role played by plants in orchestrating tritrophic interactions [30]. Future research may be oriented toward the extraction and characterization of VOCs involved in repelling herbivores and attracting natural enemies. Such research would lead to a better understanding of the phenomenon and help researchers to acquire new elements for future practical applications.

Our results highlight that in those crops where the release and subsequent conservation of *N. tenuis* is common practice (e.g., more than 80% of tomato greenhouses in southeastern Spain [41]), the persistence of this mirid on the plant throughout the growing season is an extra benefit in protecting them against pests.

In southeastern Spain, it is common practice to release *N. tenuis* adults on to the seedlings at a ratio of 0.5–1 adults per plant, and this release occurs approximately seven days before transplanting [25]. During this period, these adults—which are fed with eggs of the Mediterranean flour moth *Ephestia kuehniella* Zeller (Lepidoptera: Pyralidae)—lay eggs on the tomato plants such that when these plants are transplanted to the greenhouse, they already carry eggs of *N. tenuis*. Nevertheless, this is not the only benefit of this pre-plant release. During this seedling period, *N. tenuis* also feeds on the tomato plants, thereby activating the defenses of these plants [21]. Recently, Pappas, et al. [42], using *M. pygmaeus* as a model, proposed the term “plant vaccination” to describe this type of defensive activation induced in seedlings, because tomato plants reaching the greenhouse are “ready-to-defend” against herbivory. In the case of *M. pygmaeus*, Pappas, et al. [28] showed that this induction can last up to two weeks after the tomato plant comes in contact with the mirid. We have confirmed that this induction period is similar or longer for *N. tenuis* (same authors, unpublished data) such that the tomato plant would be “vaccinated” in the nursery, and this effect would last until the newly emerged nymphs start to feed on the plant, possibly renewing this defensive induction (as our results suggest). This effect on plants by the mirid could potentially counterbalance its potential damage on fruit [43]. The vaccination effect should be verified through field evaluations to confirm that plant activation is possible throughout the seedling, establishment, and conservation periods of *N. tenuis* in the crop.

## 4. Materials and Methods

### 4.1. Plants and Insects

Tomato plants *Solanum lycopersicum* cv. Optima (Seminis Vegetable Seeds, Inc., Almería, Spain) were sown in soil, and two weeks after germination, seedlings were individually transferred to pots (8 × 8 × 8 cm). Plants were maintained undisturbed at 25 ± 2 °C, while relative humidity and photoperiod were held constant at 65% ± 5% relative humidity and 14:10 h (Light:Dark). Pesticide-free tomato plants were used for the experiments at seven weeks of age (approximately 20 cm high). 

*B. tabaci*, *E. formosa*, and all instars and stages of *N. tenuis* were provided directly by Koppert Biological Systems, S.L. (Águilas, Murcia, Spain). Young nymphs consisted of a proportional mix of NI and NII, whereas mature nymphs corresponded to a proportional mix of NIV and NV. Adult females and males of *N. tenuis* were less than 4 days old, whereas adult females of *B. tabaci* and *E. formosa* were less than 2 days old. To obtain *N. tenuis*-punctured plants, four undamaged tomato plants were enclosed for 24 h in a 60 × 60 × 60 cm plastic cage (BugDorm-2; Mega View Science Co., Ltd., Taichung, Taiwan) and exposed to 80 *N. tenuis* of the corresponding nymphal instar or adult sex (20 individuals per plant). All motile individuals were removed from plants before the experiment. 

### 4.2. Y-Tube Bioassays

The olfactory preference of *B. tabaci* and *E. formosa* for different scent sources was tested using a Y-tube olfactometer (Analytical Research Systems, Gainesville, FL, USA). This Y-tube consisted of a Y-shaped glass tube (2.4 cm in diameter with a base of 13.5 cm in length) which was connected to two identical 5 L glass jars via plastic tubes. Each jar was connected to an air pump that produced a unidirectional airflow at a rate of 150 mL/min and contained a tested odor source (tomato plant). The experiments were conducted at 23 ± 2 °C, 60% ± 10% RH, and a light intensity of 2516 lux [44].

The first set of observations was conducted comparing the olfactory preference of *B. tabaci* and *E. formosa* adult females for each treatment of *N. tenuis*-punctured plants (young nymphs—NI and NII, mature nymphs—NIV and NV, adult males and females) relative to undamaged plants, while another series of observations was carried out comparing adult-male- versus adult-female-exposed plants and young-nymph- versus mature-nymph-exposed plants. The choice of each *B. tabaci* and *E. formosa* adult female was recorded when the insect walked a distance of 3 cm in the chosen arm; in the case that a female did not make a choice after 15 min, they were excluded from the analysis. Each individual was used only once. After testing five individuals, odor sources were interchanged to avoid any influence of asymmetries in the setup. Thirty to forty valid replicates were performed for each treatment.

### 4.3. Phytohormone Analysis

Apical parts of punctured tomato plants were exposed to *N. tenuis* at different instar stages for 24 h, and samples from undamaged plants were stored at −80 °C and analyzed to compare phytohormone concentrations. The apical part was considered to be the first 5 cm of the plant formed by the apical stem and young leaves. The phytohormone profile was analyzed using ultra-performance liquid chromatography coupled with mass spectrometry (UPLC-MS) [6,21,45]. This method can detect the concentrations of the two phytohormones involved in the tomato plant defensive responses: ABA and JA–Ile.

### 4.4. Quantification of Plant Gene Expression 

The apical parts of the plants were used to quantify the gene expression of *ASR1* (ABA stress ripening protein 1)—a marker gene for ABA—and *PIN2* (proteinase inhibitors 2), a marker gene for JA. Immediately after collection, apical samples were ground in liquid nitrogen and a portion of them served for RNA extraction. Plant RNA Kit (Omega Bio-TekInc, Doraville, GA, USA) was used to extract total RNA (1.5 μg), and RNase-free DNase (Promega Corporation, Madison, WI, USA) was employed to eliminate genomic DNA contamination. Reverse transcription, primers, and the PCR SYBR Green reaction were carried out as previously described by Pérez-Hedo, et al. [21]. Quantitative PCR was performed with the Smart Cycler II (Cepheid, Sunnyvale, CA, USA) sequence detector using standard PCR conditions. Expression of *EF1* (Elongation factor 1) was used for normalization as housekeeping gene. Table 1 describes the sequences of the gene-specific primers used.

### 4.5. Data Analyses

Data sets obtained from the Y-tube olfactometer observations were analyzed using a chi-square (χ^2^) test in order to evaluate whether the response of insects to different scent sources deviated from a null model, where odor sources were chosen with equal frequency. Data concerning gene expression levels and phytohormone analyses between different instars of *N. tenuis*-exposed plants and undamaged plants were compared by one tailed *t*-test (*p* < 0.05).

## Figures and Tables

**Table 1:** Primers used for quantification of the RNA levels of the genes studied.

Gene	Forward Primer (5’→3’)	Reverse Primer (5’→3’)
*EF1*	5-GATTGGTGGTATTGGAACTGTC-3	5-AGCTTCGTGGTGCATCTC-3
*ASR1*	5-ACACCACCACCACCACCTGT-3	5-GTGTTTGTGTGCATGTTCTGGA-3
*PIN2*	5-GAAAATCGTTAATTTATCCCAC-3	5-ACATACAAACTTTCCATCTTTA-3

## References

[1] Ton J., Flors V., Mauch-Mani B. (2009). The multifaceted role of ABA in disease resistance. Trends Plant Sci..

[2] Santino A., Taurino M., de Domenico S., Bonsegna S., Poltronieri P., Pastor V., Flors V. (2013). Jasmonate signaling in plant development and defense response to multiple (a)biotic stresses. Plant Cell Rep..

[3] Dicke M., van Loon J.J.A. (2014). Chemical ecology of phytohormones: How plants integrate responses to complex and dynamic environments. J. Chem. Ecol..

[4] Thaler J.S., Farag M.A., Pare P.W., Dicke M. (2002). Jasmonate-deficient plants have reduced direct and indirect defences against herbivores. Ecol. Lett..

[5] De Vos M., van Oosten V.R., van Poecke R.M.P., van Pelt J.A., Pozo M.J., Mueller M.J., Buchala A.J., Metraux J.P., van Loon L.C., Dicke M. (2005). Signal signature and transcriptome changes of arabidopsis during pathogen and insect attack. Mol. Plant Microbe Interact..

[6] Flors V., Ton J., van Doorn R., Jakab G., Garcia-Agustin P., Mauch-Mani B. (2008). Interplay between JA, SA and ABA signalling during basal and induced resistance against *Pseudomonas syringae* and *Alternaria brassicicola*. Plant J..

[7] Flokova K., Tarkowska D., Miersch O., Strnad M., Wasternack C., Novak O. (2014). Uhplc-ms/ms based target profiling of stress-induced phytohormones. Phytochemistry.

[8] Bari R., Jones J.D.G. (2009). Role of plant hormones in plant defence responses. Plant Mol. Biol..

[9] Kaloshian I., Walling L.L. (2005). Hemipterans as plant pathogens. Annu. Rev. Phytopathol..

[10] Howe G.A., Jander G. (2008). Plant immunity to insect herbivores. Annu. Rev. Plant Biol..

[11] Avila C.A., Arevalo-Soliz L.M., Jia L.L., Navarre D.A., Chen Z., Howe G.A., Meng Q.W., Smith J.E., Goggin F.L. (2012). Loss of function of fatty acid desaturase7 in tomato enhances basal aphid resistance in a salicylate-dependent manner. Plant Physiol..

[12] Alba J.M., Schimmel B.C.J., Glas J.J., Ataide L.M.S., Pappas M.L., Villarroel C.A., Schuurink R.C., Sabelis M.W., Kant M.R. (2015). Spider mites suppress tomato defenses downstream of jasmonate and salicylate independently of hormonal crosstalk. New Phytol..

[13] Nalam V.J., Keeretaweep J., Sarowar S., Shah J. (2012). Root-derived oxylipins promote green peach aphid performance on arabidopsis foliage. Plant Cell Online.

[14] Heil M. (2008). Indirect defence via tritrophic interactions. New Phytol..

[15] Coppola V., Coppola M., Rocco M., Digilio M.C., D’Ambrosio C., Renzone G., Martinelli R., Scaloni A., Pennacchio F., Rao R. (2013). Transcriptomic and proteomic analysis of a compatible tomato-aphid interaction reveals a predominant salicylic acid-dependent plant response. BMC Genom..

[16] Ponzio C., Gols R., Pieterse C.M.J., Dicke M. (2013). Ecological and phytohormonal aspects of plant volatile emission in response to single and dual infestations with herbivores and phytopathogens. Funct. Ecol..

[17] Halitschke R., Hamilton J.G., Kessler A. (2011). Herbivore-specific elicitation of photosynthesis by mirid bug salivary secretions in the wild tobacco *Nicotiana attenuata*. New Phytol..

[18] Kessler A., Baldwin I.T. (2004). Herbivore-induced plant vaccination. Part I. The orchestration of plant defenses in nature and their fitness consequences in the wild tobacco Nicotiana attenuata. Plant J..

[19] De Puysseleyr V., Hofte M., de Clercq P. (2011). Ovipositing *Orius laevigatus* increase tomato resistance against *Frankliniella occidentalis* feeding by inducing the wound response. Arthropod-Plant Interact..

[20] Pérez-Hedo M., Bouagga S., Jaques J.A., Flors V., Urbaneja A. (2015). Tomato plant responses to feeding behavior of three zoophytophagous predators (hemiptera: Miridae). Biol. Control..

[21] Pérez-Hedo M., Urbaneja-Bernat P., Jaques J.A., Flors V., Urbaneja A. (2015). Defensive plant responses induced by *Nesidiocoris tenuis* (hemiptera: Miridae) on tomato plants. J. Pest Sci..

[22] Perdikis D., Lykouressis D.P., Economou L.P. (1999). The influence of temperature, photoperiod and plant type on the predation rate of *Macrolophus pygmaeus* on *Myzus persicae*. BioControl.

[23] Sanchez J.A., Gillespie D.R., McGregor R.R. (2004). Plant preference in relation to life history traits in the zoophytophagous predator *Dicyphus hesperus*. Entomol. Exp. Appl..

[24] Urbaneja A., Tapia G., Stansly P. (2005). Influence of host plant and prey availability on developmental time and surviorship of *Nesidiocoris tenius* (het.: Miridae). Biocontrol Sci. Technol..

[25] Calvo F.J., Bolckmans K., Belda J.E. (2012). Release rate for a pre-plant application of *Nesidiocoris tenuis* for *Bemisia tabaci* control in tomato. BioControl.

[26] Zappalà L., Biondi A., Alma A., Al-Jboory I.J., Arnò J., Bayram A., Chailleux A., El-Arnaouty A., Gerling D., Guenaoui Y. (2013). Natural enemies of the South American moth, *Tuta absoluta*, in Europe, North Africa and Middle-East, and their potential use in pest control strategies. J. Pest Sci..

[27] Zappala L., Siscaro G., Biondi A., Molla O., Gonzalez-Cabrera J., Urbaneja A. (2012). Efficacy of sulphur on *Tuta absoluta* and its side effects on the predator *Nesidiocoris tenuis*. J. Appl. Entomol..

[28] Pappas M.L., Steppuhn A., Geuss D., Topalidou N., Zografou A., Sabelis M.W., Broufas G.D. (2015). Beyond predation: The zoophytophagous predator *Macrolophus pygmaeus* induces tomato resistance against spider mites. PLoS ONE.

[29] Calvo J., Blockmans K., Stansly P.A., Urbaneja A. (2009). Predation by *Nesidiocoris tenuis* on *Bemisia tabaci* and injury to tomato. BioControl.

[30] Fatouros N.E., Lucas-Barbosa D., Weldegergis B.T., Pashalidou F.G., van Loon J.J.A., Dicke M., Harvey J.A., Gols R., Huigens M.E. (2012). Plant volatiles induced by herbivore egg deposition affect insects of different trophic levels. PLoS ONE.

[31] Heil M., Silva Bueno J.C. (2007). Within-plant signaling by volatiles leads to induction and priming of an indirect plant defense in nature. Proc. Natl. Acad. Sci. USA.

[32] Colazza S., McElfresh J.S., Millar J.G. (2004). Identification of volatile synomones, induced by *Nezara viridula* feeding and oviposition on bean spp., that attract the egg parasitoid trissolcus basalis. J. Chem. Ecol..

[33] Kahn T.L., Fender S.E., Bray E.A., Oconnell M.A. (1993). Characterization of expression of drought and abscisic acid-regulated tomato genes in the drought-resistant species *Lycopersicon pennellii*. Plant Physiol..

[34] Maskin L., Gudesblat G.E., Moreno J.E., Carrari F.O., Frankel N., Sambade A., Rossi M., Iusem N.D. (2001). Differential expression of the members of the *ASR* gene family in tomato (*Lycopersicon esculentum*). Plant Sci..

[35] Ramirez V., Coego A., Lopez A., Agorio A., Flors V., Vera P. (2009). Drought tolerance in arabidopsis is controlled by the ocp3 disease resistance regulator. Plant J..

[36] Kessler A., Baldwin I.T. (2002). Plant responses to insect herbivory: The emerging molecular analysis. Annu. Rev. Plant Biol..

[37] Abe H., Shimoda T., Ohnishi J., Kugimiya S., Narusaka M., Seo S., Narusaka Y., Tsuda S., Kobayashi M. (2009). Jasmonate-dependent plant defense restricts thrips performance and preference. BMC Plant Biol..

[38] Agut B., Gamir J., Jacas J.A., Hurtado M., Flors V. (2014). Different metabolic and genetic responses in citrus may explain relative susceptibility to *Tetranychus urticae*. Pest Manag. Sci..

[39] Agut B., Gamir J., Jaques J.A., Flors V. (2015). *Tetranychus urticae*-triggered responses promote genotype-dependent conspecific repellence or attractiveness in citrus. New Phytol..

[40] Dicke M. (2009). Behavioural and community ecology of plants that cry for help. Plant Cell Environ..

[41] Pérez-Hedo M., Urbaneja A., Horowitz A.R., Ishaaya I. (2016). The zoophytophagous predator *Nesidiocoris tenuis*: A successful but controversial biocontrol agent in tomato crops. Advances in Insect Control and Resistance Management.

[42] Pappas M.L., Steppuhn A., Broufas G.D. (2016). The role of phytophagy by predators in shaping plant interactions with their pests. Commun. Integr. Biol..

[43] Biondi A., Zappala L., di Mauro A., Garzia G.T., Russo A., Desneux N., Siscaro G. (2016). Can alternative host plant and prey affect phytophagy and biological control by the zoophytophagous mirid *Nesidiocoris tenuis*?. BioControl.

[44] Pérez-Hedo M., Urbaneja A. (2015). Prospects for predatory mirid bugs as biocontrol agents of aphids in sweet peppers. J. Pest Sci..

[45] Forcat S., Bennett M.H., Mansfield J.W., Grant M.R. (2008). A rapid and robust method for simultaneously measuring changes in the phytohormones ABA, JA and SA in plants following biotic and abiotic stress. Plant Methods.

